# RKIP: A Pivotal Gene Product in the Pathogenesis of Cancer

**DOI:** 10.3390/cancers13102488

**Published:** 2021-05-20

**Authors:** Benjamin Bonavida

**Affiliations:** Department of Microbiology, Immunology and Molecular Genetics, David Geffen School of Medicine, College of Life Sciences, UCLA, Los Angeles, CA 90095, USA; bbonavida@mednet.ucla.edu

Since its original cloning by Yeung et al. [1], investigations on the Raf Kinase Inhibitor Protein (RKIP) in normal individuals, several diseases, and particularly in various cancers have been steadily expanding concurrently with an increasing number of publications. RKIP is intimately involved in the pathogenesis of many cancers, as the majority of cancers express very low levels of RKIP. RKIP has been reported as a tumor suppressor [2], an immune enhancer [3], a prognostic/diagnostic biomarker [4], and a therapeutic target [5].

The epi/genetic, molecular, and biochemical analyses of the underlying mechanisms of the regulation and function of RKIP in cancer have covered a spectrum of topics, which include the general properties of RKIP in human malignancies, its regulation by phosphorylation [6] and micro-RNAs [7], signaling cross-talks [8], gene products that are regulated by RKIP [9] or genes products that regulate RKIP expression in various cancers [10], the pleiotropic functional activities of RKIP in cancer (roles in proliferation, survival, EMT [11], chemo-radio-immunoresitance [12], autophagy [13], etc.), the response to photo-oxidative damage [14], role in hypoxia and cellular stress [15], cellular plasticity [16], role in inflammation [17], role as a prognostic/ diagnostic marker, and new agents as therapeutic targets that are directed against RKIP, used alone or in combination, in the treatment of resistant cancers to conventional therapies [12].

Several reports have described the role that RKIP plays in many cancers. The majority of human cancers express very low levels of RKIP when compared to adjacent normal tissues. However, there are a few instances where RKIP is overexpressed, but in its inactive phosphorylated form, such as the case in multiple myeloma [8]. Among the many hallmarks of cancer, signaling modules (namely, p53, STAT-3, nuclear factor κB (NF-κB), and SNAIL) suggest the novel roles for RKIP in the control of autophagy and vice versa. RKIP and the microtubule-associated protein 1 light chain 3 (MAP1LC3, LC3) in autophagy regulate cell proliferation, senescence, and the epithelial to mesenchymal transition (ETM) [18,19]. It is noteworthy that there are instances whereby RKIP expression is absent, like in acute myeloid leukemia (AML) and other myeloid neoplasias. Preclinical findings suggest that RKIP is pivotal in the development of these non-solid tumors [19].

In light of the well-recognized pleiotropic role of RKIP in the pathogenesis of cancer and its new implications in the prognosis of various cancers and the regulation of both cancer malignancies and the resistance to chemo-immuno-therapeutis, for this Special Issue, established researchers are invited to contribute original articles or reviews that report their latest findings or literature data regarding the biochemical, epi/genetic, molecular, and physiological underlying mechanisms of RKIP regulation and activity, and particularly various means of its selective induction in cancers with the ultimate designs and developments of new therapeutic agents that function to inhibit malignancies and resistance.
